# Strain-Insensitive Conductive Hydrogel Materials for Motion-Artifact-Free Flexible Bioelectronics

**DOI:** 10.3390/gels12090822

**Published:** 2026-09-07

**Authors:** Yarong Ding, Yitong Dou, Lei Bai, Zhenyu Li, Jiayi Qi, Yufeng Li, Shaozhe Tan, Xuesi Zhang, Jiachun Sun, Yahui Song, Jingxuan Wu, Fei Han, Yingchun Li

**Affiliations:** 1 State Key Laboratory of Wide-Bandgap Semiconductor Devices and Integrated Technology, Faculty of Integrated Circuit, Xidian University, Xi’an 710071, China24149100095@stu.xidian.edu.cn (Y.D.); 24069100120@stu.xidian.edu.cn (L.B.); 22009102081@stu.xidian.edu.cn (Z.L.); qjy33567368@163.com (J.Q.); akanarikanotan@foxmail.com (S.T.); 15395679021@163.com (X.Z.); 22009101666@stu.xidian.edu.cn (J.S.); syahui1201@163.com (Y.S.); 13428816759@163.com (J.W.); 2 Shaanxi Key Laboratory of Degradable Biomedical Materials, School of Chemical Engineering, Northwest University, Xi’an 710069, China; 15729515001@163.com; 3 School of Life Science and Technology, Xi’an Jiaotong University, Xi’an 710049, China; 4 Advanced Interdisciplinary Research Center for Flexible Electronics, Faculty of Infor-X, Xidian University, Xi’an 710071, China

**Keywords:** strain insensitivity, conductive hydrogels, bioelectronics

## Abstract

Flexible and stretchable electronics inevitably undergo stretching, compression, bending and torsion when conformally attached to skin, soft tissues and dynamic organs. While deformation-induced electrical variations act as target signals for motion sensors, they cause resistance/impedance drift, baseline shift and sensitivity degradation in physiological electrodes, temperature/chemical sensors, interconnects and stimulation devices, leading to motion artifacts and reduced long-term reliability. Hydrogels are pivotal materials for soft bioelectronic interfaces owing to their high water content, low modulus, tissue compatibility and ionic conductivity. However, their conductive networks are susceptible to structural reconstruction under deformation, dehydration, swelling and cyclic fatigue, meaning that stretchability is by no means equivalent to strain insensitivity. This review focuses on stable resistance/impedance and functional output within a specified strain window, this paper reviews three representative material systems, liquid metal (LM)-based composite hydrogels, conductive polymer/elastic network composite hydrogels, and hydrogen-bonded isotropic architectures. It further summarizes three design strategies—geometric and functional compensation, mechanical decoupling and strain isolation, and interfacial engineering for conductive network stabilization—and discusses their applications in wearable epidermal and implantable bioelectronics. Finally, unified evaluation metrics for strain insensitivity are proposed, with future directions covering high-conductivity–low-modulus synergy, long-term water/ionic stability, robust soft-hard interfaces, multiaxial deformation tolerance and scalable manufacturability.

## 1. Introduction

Flexible electronics demonstrate tremendous application potential in health monitoring, human–machine interaction, implantable medicine and other fields, benefiting from their tissue-matched mechanical properties and superior conformal interfacial adhesion [1,2,3,4]. As wearable and implantable devices advance toward high integration and high stability, dynamic deformation interference encountered by devices during service has gradually emerged as a core bottleneck [5]. Physiological movements of human skin and organs (e.g., pulse pulsation, joint bending, cardiac beating) exert a wide range of strain loads on electronic devices, causing fracture and reconstruction of conductive pathways in conventional conductive materials [6]. This, in turn, triggers resistance drift, signal distortion and even functional failure, severely limiting the detection accuracy and long-term reliability of devices [7,8].

Conductive hydrogels are a class of soft matter materials constructed by hydrophilic polymer networks composited with conductive media (ions, conductive fillers, and conductive polymers) [9,10,11]. They integrate tissue-like low elastic modului (1–100 kPa), favorable biocompatibility, tunable mechanical properties and ionic/electronic conductivity, and they are recognized as ideal substrate materials for next-generation bio-integrated electronics [12,13]. Compared with traditional elastomer-based conductors, the water-rich nature and three-dimensional porous network structure of hydrogels enable better matching with the mechanical and chemical environments of biological tissues, endowing them with inherent advantages in scenarios such as epidermal physiological signal acquisition and implantable neural interfaces [14,15].

For homogeneous conductors, resistance can be approximated as R = ρL/A. Uniaxial stretching increases the length L, reduces the cross-sectional area A, and may alter the resistivity ρ [16]. For filler-type conductive hydrogels, deformation also modifies interparticle distance, tunneling barrier, lamellar overlap, orientation of conductive polymer chains and ionic concentration distribution. Therefore, even without material fracture, resistance or impedance may drift significantly, eventually causing pronounced fluctuations in electrical conductivity [17,18], which fails to meet the demand for stable signal transmission under dynamic deformation [19]. Strain sensors precisely leverage such variations, whereas physiological electrodes, temperature sensors, chemical sensors and interconnects generally require such variations to be minimized [20,21]. Accordingly, developing strain-insensitive conductive hydrogels to achieve stable output of electrical properties over a wide strain range has become a critical research direction in flexible electronics.

**Figure 1:**
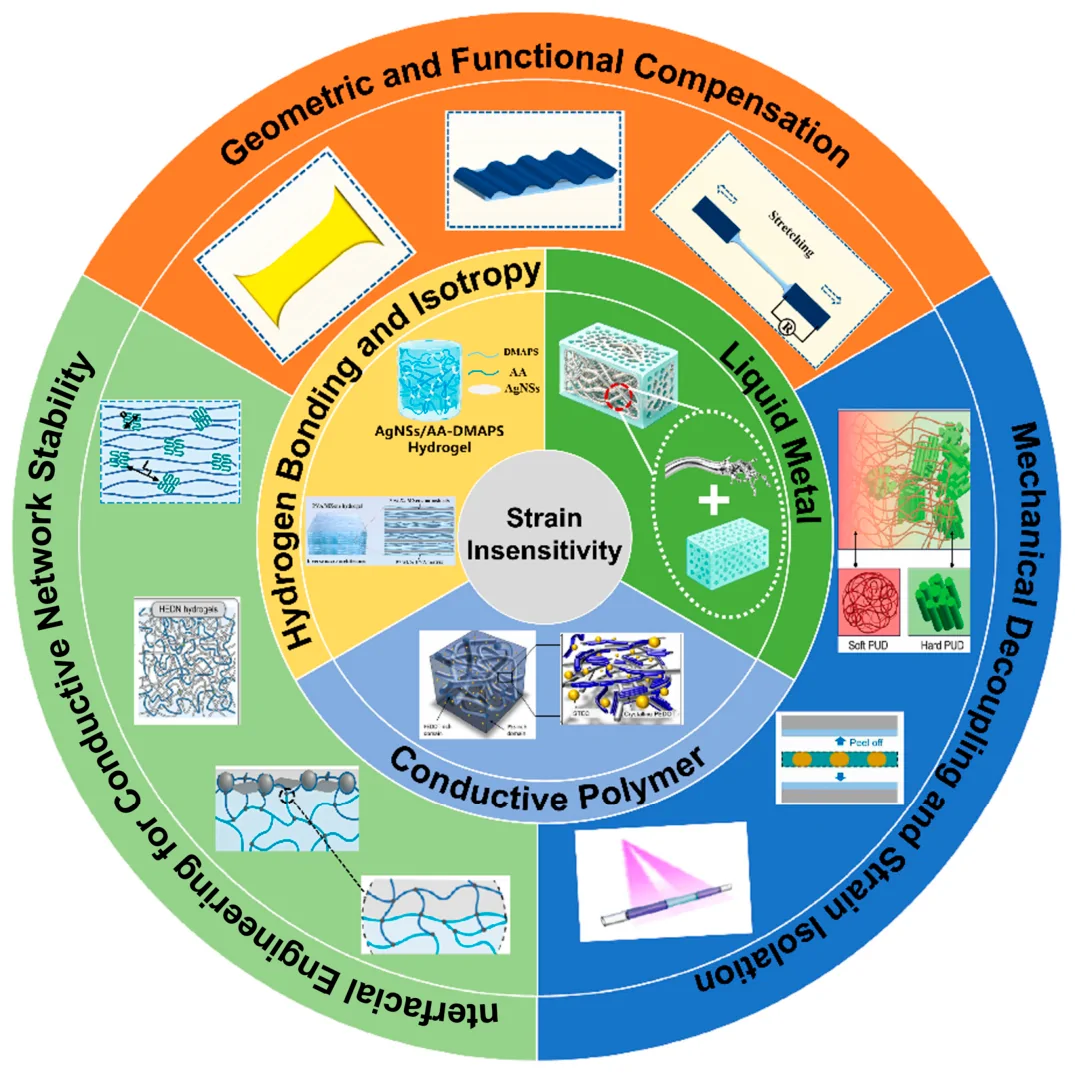
Overview of solution strategies for strain-insensitive conductive hydrogel materials. Centered on “strain insensitivity”, this diagram presents the intrinsic regulation mechanisms of three core material systems: LM-based composite hydrogels, conductive polymer/elastic network composite hydrogels, and hydrogen-bonded isotropic architectures. It further delineates device-level implementation strategies, including geometric and functional compensation (wrinkled, buckled, and serpentine structures), mechanical decoupling and strain isolation (island-bridge configurations and layered architectures), and interfacial engineering for conductive network stabilization. The diagram elucidates how each material system can be synergistically coupled with one or more of these strategies to maintain stable electrical output under mechanical deformation. Reproduced with permission [22]; Copyright 2020, Wiley-VCH Verlag. Reproduced with permission [23]; Copyright 2026, Wiley-VCH GmbH. Reproduced with permission [24]; Copyright 2017, American Association for the Advancement of Science. Reproduced with permission [25]; Copyright 2025, Wiley-VCH GmbH. Reproduced with permission [26]; Copyright 2026, Springer Nature. Reproduced with permission [27]; Copyright 2021, American Association for the Advancement of Science. Reproduced with permission [28]; Copyright 2025, Wiley-Blackwell. Reproduced with permission [29]; Copyright 2026, Wiley-VCH GmbH. Reproduced with permission [30]; Copyright 2022, Institute of Physics and IOP Publishing Limited 2024. Reproduced with permission [31]; Copyright 2024, Springer Nature.

The core of strain-insensitive conductive hydrogels is that key electrical parameters such as conductivity and impedance remain relatively stable within a given strain range, with the relative resistance change rate far below the theoretical prediction of conventional conductors [7]. Starting from material systems and intrinsic regulation mechanisms, this paper systematically reviews the research progress of three categories of strain-insensitive hydrogels: LM-based systems, conductive polymer-based systems, and hydrogen-bonding network-tailored systems. Furthermore, from the perspective of device engineering, three implementation strategies are generalized: geometric and functional compensation, mechanical decoupling and strain isolation, and interfacial engineering and network stabilization (Figure 1). Finally, their typical applications in wearable epidermal electronics and implantable bioelectronics are introduced, and the current challenges and future development directions are summarized and prospected.

## 2. Core Material Systems

### 2.1. LM-Based Composite Hydrogel Materials

LMs (e.g., gallium–indium–tin alloys) combine the high electrical conductivity of metals with the intrinsic fluidity of liquids, making them ideal functional fillers for constructing strain-insensitive conductors [32,33]. Dispersing LM microdroplets into a hydrogel matrix yields a composite system with coexisting electron-conducting phase and aqueous polymer phase [34]. The advantage of LMs is that the conductive phase itself does not undergo brittle fracture, and its local rearrangement can compensate for changes in length and cross-sectional area. Their inherent flow, coalescence, and self-healing properties of conductive pathways under deformation are exploited to counteract the destructive effect of strain on the conductive network [35]. However, simply dispersing LM microdroplets uniformly in hydrogels or elastomers usually requires mechanical sintering or high filler loading to form continuous pathways and may suffer from leakage, migration, and cyclic fatigue [36,37].

**Figure 2:**
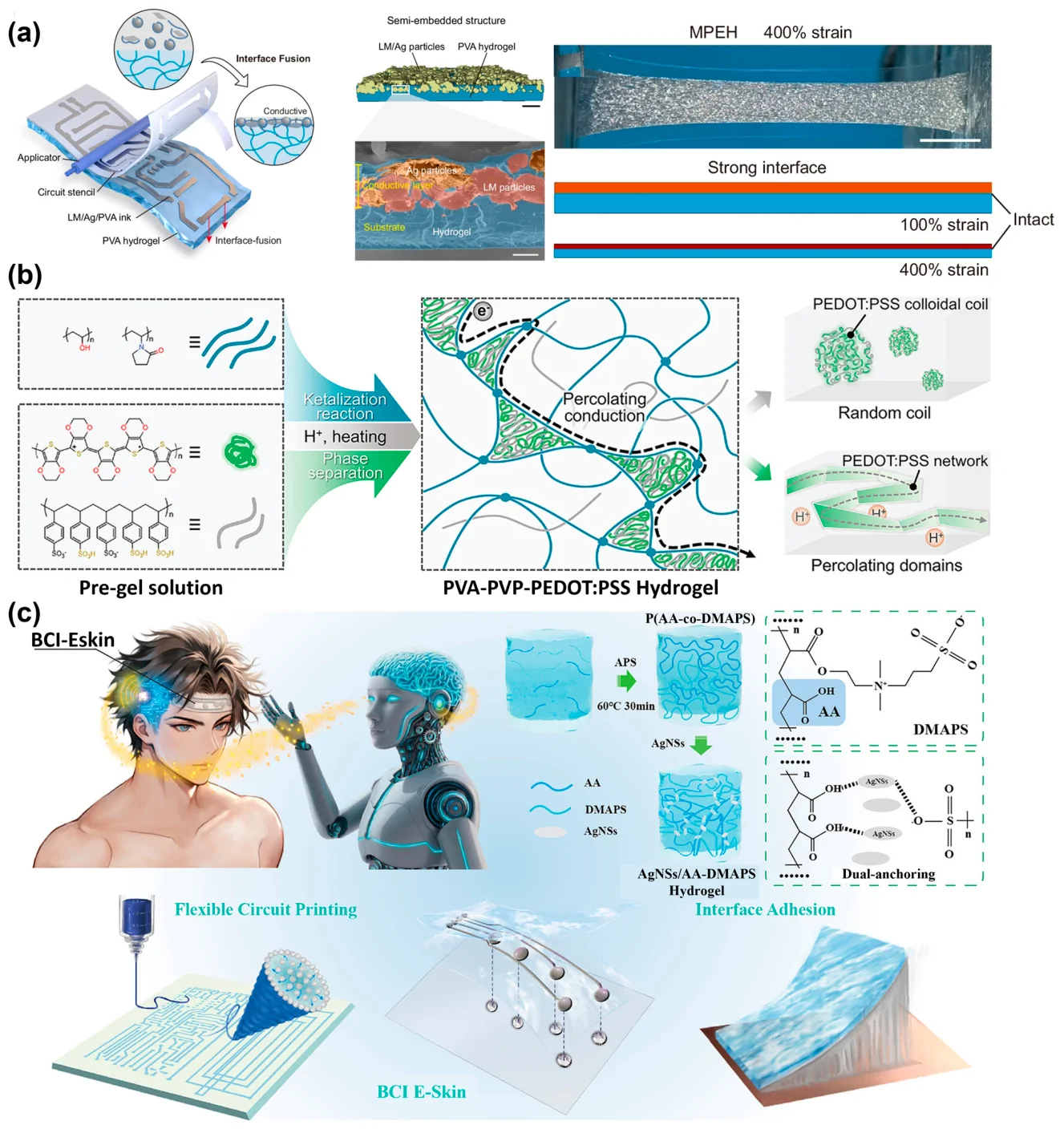
(**a**) Schematic illustration of the components and the fabrication process of this work. The flexible mask used for patterning was fabricated from 300 µm thick thermoplastic polyurethane via laser engraving, and micro-computed tomography (micro-CT) images show the semi-embedded structure of liquid metal (LM)/Ag particles in this work. Scale bar, 400 µm; cross-sectional SEM image showing seamless integration of the LM/Ag circuit with the PVA hydrogel substrate, with no visible gap. The LM/Ag particles form a stable conductive pathway. Scale bar, 50 µm; optical images of the LM/Ag/PVA conductive layer on different substrates before and after stretching. Scale bar, 1 cm. Reproduced with permission: Copyright 2026, Springer Nature [26]. (**b**) Schematic illustration of one-step acid-induced preparation of PVA-PVP-PEDOT:PSS bicontinuous-phase conductive hydrogel. Reproduced with permission: Copyright 2024, Wiley-VCH [46]. (**c**) Schematic diagram of silver nanosheet (AgNS)/AA-DMAPS preparation and application principles. Reproduced with permission: Copyright 2026, Wiley-VCH [23].

One effective approach is to construct a “solid–liquid synergistic” bicontinuous network [38]. Rigid silver microflakes, silver nanowires, or copper particles are responsible for spanning large spatial scales and providing stable overlaps, while LMs fill microcracks and maintain dynamic contacts [39,40]. Furthermore, interfacial bonding strength is a core factor determining the strain stability of LM composite hydrogels. As shown in Figure 2a, Jiao et al. developed an interfacially fused LM–hydrogel composite, which alleviates stress concentration during stretching by enhancing interfacial interactions and preserves the structural integrity of the conductive layer [26]. The highly durable metallogel constructed by Li et al. immobilizes the continuous LM phase with a waterborne polyurethane network. Combined with dynamic hydrogen bonding and reversible polymer chain orientation mechanisms, it maintains high electrical conductivity after one million cyclic stretches, and the conductive network fully recovers to its initial isotropic state upon strain release [41]. Layered gravity-driven LM-doped hydrogels further enhance the continuity of conductive pathways under deformation through composition gradient design. Zhang et al. proposed interfacial fusion printing, in which interconnected LM/silver particles are semi-embedded into the hydrogel’s surface [42]. The results show that the R/R_0_ of different hydrogel substrates remains at a low increase under large stretching. This also indicates that it is not the LM itself that is prone to fracture, but rather the slippage, wrinkling, and leakage that readily occur between the LM composite layer and the high-water-content matrix.

### 2.2. Conductive Polymers and Hydrogel Matrices

Intrinsically conductive polymers such as poly(3,4-ethylenedioxythiophene):polystyrene sulfonate (PEDOT:PSS), polyaniline, and polypyrrole exhibit excellent electronic conductivity and electrochemical stability [6,24]. However, their rigid molecular chains and high intrinsic brittleness give rise to inherent thermodynamic incompatibility with hydrophilic hydrogel matrices [43]. During deformation, the conductive polymer phase is prone to phase separation from the hydrogel network and brittle fracture of conductive pathways, ultimately leading to severe nonlinear drifts in resistance and impedance. This is the core cause of strain sensitivity in conventional conductive polymer hydrogels [12,44]. The key to addressing this issue lies in locking the conductive phase and the load-bearing network across multiple scales.

At the molecular scale, strain insensitivity can be achieved while enhancing adhesion via interpenetrating networks, in situ polymerization, and dynamic bonds [45]. Han et al. constructed a bicontinuous conductive percolation network of PEDOT:PSS through one-step acid-induced in situ phase separation [46]. The π-π stacking between PEDOT chains and the hydrogen-bonded interface with the PVA/PVP (polyvinyl alcohol/polyvinyl pyrrolidone) matrix stabilizes the electronic conductive pathways. The conductive network remains intact in the low-strain regime, effectively suppressing resistance drift under stretching and enabling motion-insensitive sensing (Figure 2b). Additionally, conductive monomers or oligomers infiltrating into the surface layer of elastic fibers/hydrogels followed by in situ polymerization can form chain entanglements and dual chemical/physical anchoring [47]. Liang et al. proposed an “interfacial confinement locking” strategy spanning from the nanoscale to the macroscale: the conductive polymer forms molecular entanglements with the substrate while being confined within the pores of the electrospun membrane, maintaining nearly constant resistance within approximately 200% strain [48].

At the macroscopic level, Janus adhesion and selective bonding can be exploited. Liu et al. fabricated Janus organohydrogels with rigid-flexible interlocked conductive networks and spatially separated metal phases via gravity inversion annealing. These gels maintain high conductivity under large strain while exhibiting a pronounced difference in bilateral adhesion [49]. Such designs integrate strain stability, motion artifact resistance, and interfacial positioning, representing an important development direction for hydrogel bioelectronics [50,51].

## 3. Intrinsic Regulation Mechanisms

### 3.1. Regulation of Hydrogen-Bonding Networks

The strain dependence of hydrogels largely arises from chain-segment orientation, hydrogen bond breakage/reformation, and water migration. Li et al. developed 3D-printable silver nanosheet/amphoteric copolymer hydrogels, in which interchain hydrogen bonds between acrylic acid (AA) and [2-(Methacryloyloxy)ethyl]dimethyl-(3-sulfopropyl)ammonium hydroxide (DMAPS) are utilized to construct a uniform polymer matrix [23]. Silver nanosheets form an orientation-free continuous conductive network inside the gel via dual-anchoring coordination interactions. During stretching, hydrogen bonds and dynamic Ag coordination bonds reversibly break and re-form to stabilize conductive pathways, resulting in minimal resistance change within 500% elongation, along with ultrahigh conductivity and strong adhesion to multiple substrates (Figure 2c).

### 3.2. Isotropic Conductive Networks

Although conventional unidirectional freeze-casting, strain-induced orientation, or striped microstructures can improve performance in a single direction, they may induce pronounced anisotropy [6]. The electrical responses differ along and perpendicular to the texture direction, making it difficult to withstand multiaxial human motion. Accordingly, constructing statistically isotropic polymer networks and surface structures is a critical pathway to achieving biaxial strain insensitivity [47].

The core of isotropic design includes in-plane network topology with no preferred orientation, which can be realized via uniform crosslinking or hierarchical interpenetration [25]. The Hofmeister effect provides a chemical tool for regulating polymer–water–ion interactions. The salting-out effect of specific anions can promote polymer chain aggregation and crystallization, reshape hydrogen-bonding networks, and increase network density and energy dissipation. Wang et al. employed the Hofmeister effect to regulate hydroxypropyl cellulose (HPC)/PVA cellulose-based networks, forming randomly oriented isotropic internal networks and constructing uniform micropyramid arrays on the surface [52].

Table 1 systematically summarizes the core material systems and intrinsic regulation mechanisms of strain-insensitive conductive hydrogels. The two categories of regulatory logics act synergistically, spanning from material component selection to microscale network topology optimization, and provide a hierarchical design pathway for constructing flexible conductive hydrogels with high stability and multiaxial deformation resistance.

## 4. Core Implementation Strategies for Strain-Insensitive Hydrogel Devices

### 4.1. Geometric and Functional Compensation Strategy

This strategy absorbs external deformation through geometric morphological changes in conductive networks, device units or functional media or actively offsets the impact of strain on device performance by leveraging synergistic variations in intrinsic material parameters. It is divided into two pathways: geometric deformation absorption and functional response compensation [7].

The most straightforward regulation approach for geometric deformation absorption is to fabricate the hydrogel conductive layer into stretchable geometric structures, such as serpentine, buckled, and wavy configurations, so that macroscopic strain is mainly dissipated through structural unfolding and deformation rather than acting on the conductive material itself [53]. Bi et al. adopted a planar geometric structure consisting of self-assembled wrinkled conductive rubber electrodes and polyacrylamide (PAM)/NaCl ionic hydrogels [54]. The wrinkled configuration unfolds layer by layer under stretching; the change in the effective conductive length of the electrode is offset by the geometric deformation of the wrinkles, leaving the capacitive signal unaffected by stretching. Meanwhile, the thermal and mechanical responses of the ionogel exhibit naturally distinct temporal characteristics. For the pre-strain buckling design, a pre-stretch is first applied to the elastic substrate, followed by attachment of the hydrogel functional layer; upon release of the pre-strain, the functional layer forms a micro-buckled structure [51]. When the device is subjected to stretching, the buckled structure is first flattened, and the strain actually borne by the functional layer is far lower than the macroscopic strain, thereby stabilizing electrical performance [8]. Sun et al. constructed a semi-interpenetrating double-network hydrogel based on PVA-PEDOT:PSS. Under stretching, the PVA elastic skeleton deforms and absorbs strain energy, while only local micro-fracture occurs in the PEDOT conductive fragments [55]. Dynamic hydrogen bonds clamp the conductive pathways and inhibit the overall disconnection of the conductive network, resulting in only a slight increase in resistance change at 100% strain.

Functional response compensation utilizes changes in other physical parameters of the material under deformation to reversely compensate for performance drift caused by strain [56]. For example, Xie et al. designed a zoned geometric hydrogel dual-modal sensor. The geometric-functional compensation effect of the wavy wiring offsets the impact of tensile deformation on the temperature-sensing channel, enabling simultaneous, crosstalk-free detection of temperature and strain signals and solving the problem of motion interference in temperature measurement for wearable devices [57]. This principle actively compensates for strain interference through the deformation response of functional media, breaking through the traditional design framework of “passively bearing deformation” [7].

### 4.2. Mechanical Decoupling and Strain Isolation Strategy

The mechanical decoupling strategy separates external macroscopic deformation from the strain borne by local functional regions through device-level design and mechanical path regulation [27], keeping key electronic components in a low-strain or near-zero-strain state and eliminating the interference of strain on performance from the mechanical root cause [55].

The layered decoupling architecture is the most widely applied multi-layer design featuring an “elastic support layer-buffer layer-functional layer” configuration. The outer highly elastic hydrogel matrix bears the majority of deformation, and the intermediate low-modulus buffer layer dissipates stress through its own deformation, greatly attenuating strain in the inner core functional region [28]. For instance, in the design of implantable neural electrodes, a hydrogel–elastomer composite layered structure can dissipate macroscopic strain on the body surface or organ surface layer by layer, confining the strain in the electrode functional region to within 20% and ensuring long-term stability of interfacial impedance [15].

**Figure 3:**
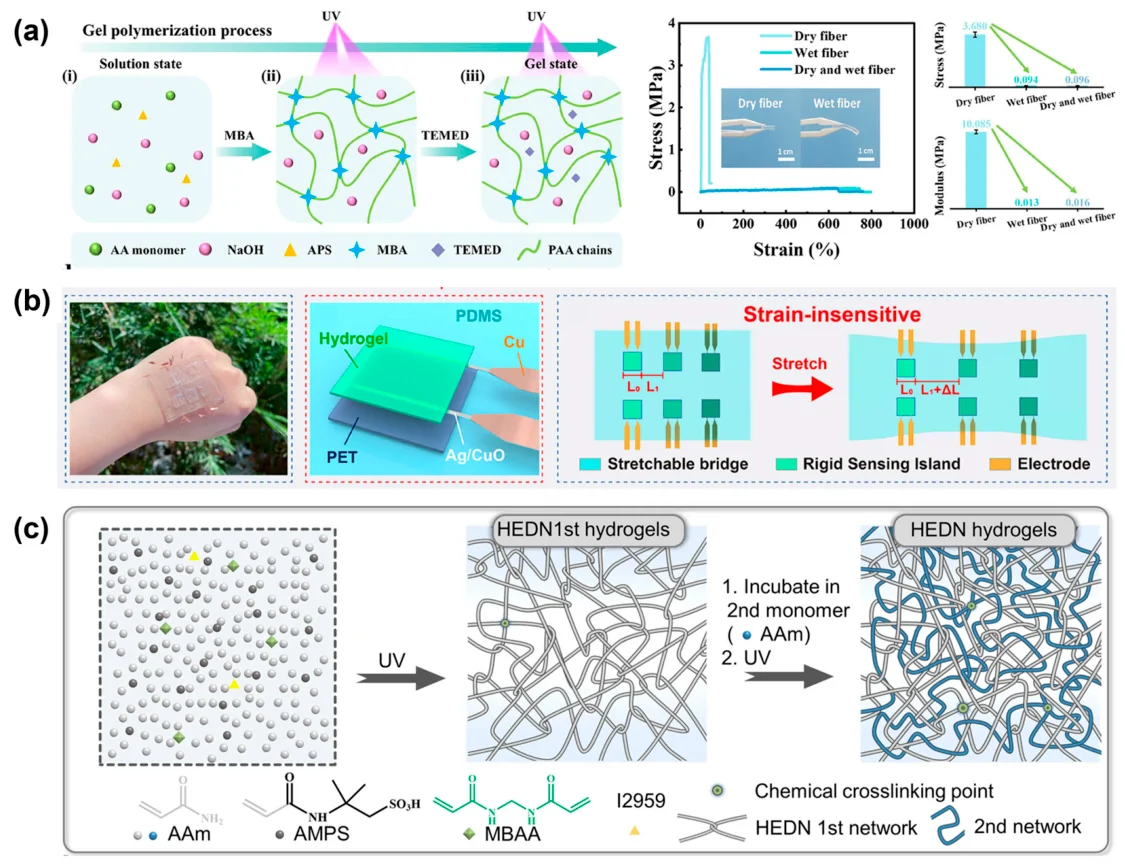
(**a**) Polymerization process of island-bridge integrated multi-functional gel fibers, as well as the stress-strain curves and modulus comparison diagram of dry fibers, wet fibers, and dry-wet composite fibers on the right. Reproduced with permission: Copyright 2026, Wiley-VCH [29]. (**b**) Photographs of SSIM electronic skin, sensing unit structure and strain-insensitive mechanism: the size of sensing islands remains unchanged before and after stretching. Reproduced with permission: Copyright 2025, Wiley-VCH [59]. (**c**) Schematic of the fabrication method for HEDN hydrogel. First, the HEDN1st hydrogel is synthesized via UV-initiated free-radical copolymerization of AAm and AMPS. Subsequently, the HEDN1st hydrogel is immersed in the second-network monomer solution for 24 h. Finally, the second network is formed in situ to obtain the HEDN hydrogel. Reproduced with permission: Copyright 2024, Springer Nature [31].

Strain distribution management and physical isolation achieve strain avoidance through local mechanical regulation. Stress concentration structures or rigid isolation units are arranged around the functional region to guide strain transfer toward non-functional areas [58]. Chen et al. fabricated “island-bridge” integrated multifunctional gel fibers by regulating the modulus via dry-wet zoning [29]. Rigid xerogel islands serve as the temperature and humidity sensing functional region, while flexible moisturized hydrogel bridges bear all tensile deformation. Physical strain isolation is realized through mechanical zoning so that temperature and humidity signals are free from interference by limb stretching (Figure 3a). Luo et al. formed an island-bridge structure via chemical anchoring treatment with poly(ethylene dimethyl terephthalate) silane, constructing multi-regional ion conduction pathways (Figure 3b). Mechanical strain isolation is achieved through differentiated design of mechanical modulus in different regions within the gel. Under stretching, stress is only dissipated in the flexible gel buffer zone, and the ionic conductive network does not fracture or reconstruct due to stretching, achieving an advanced low strain interference of 0.2% (0–55%) [59].

### 4.3. Interfacial Engineering and Conductive Network Stabilization Strategy

Interfaces are high-incidence areas for electrical failure under strain. Interfacial engineering improves adhesion stability, conductive pathway continuity, and cyclic reliability during deformation by regulating the interfacial interactions between hydrogel functional materials and elastic substrates, between different functional layers, and at electrode contact regions [60].

Interlayer interfacial regulation is realized by introducing active functional groups on the surface of conductive fillers to form dynamic interactions such as hydrogen bonds and coordination bonds with hydrogel polymer chains [61]. This not only improves filler dispersibility but also restricts excessive slippage of fillers during deformation, maintaining the connectivity of the conductive network [62]. Liu et al. utilized the hydroxyl and carboxyl-rich active functional groups on cellulose nanofiber (CNF) surfaces to construct a hydrogen-bonded interlayer interfacial regulation network with PVA molecular chains, forming ordered laers through-going ion channels [63]. This eutectogel exhibits tensile strain insensitivity (GF = 0.11, 0–400%) along the direction of oriented freezing. Perpendicular to the orientation direction, the material achieves high-sensitivity piezoresistive sensing (S = 39.02 kPa^−1^, 0.5–3.5 kPa) and maintains stable pressure signal output even under 80% disturbing strain and bending loads. Zhao et al. prepared silver microflake/LM (EGaIn) composite PVA organic self-healing conductive gels. Mechanical self-healing is achieved through the reversible hydrogen-bonded matrix formed by PVA and borate; meanwhile, a dry annealing process promotes the formation of a continuous conductive percolation network between silver microflakes and LM fillers at the gel interlayers. The dynamic interfacial interaction between fillers and polymer chains alleviates the fracture of conductive pathways caused by stretching. The material can withstand stretching of over 400%, with a conductivity recovery rate of 95% [62].

Double-network ionic hydrogels incorporate dynamic and reversible ionic crosslinking sites; under stretching, ionic crosslinks reversibly break and re-form to maintain continuous ion transport channels [64]. A highly entangled Acrylamide/2-Acrylamide-2-methylpropanesulfonic acid (AAm/AMPS)-based double-network hydrogel with high-density physical entanglements as the core was fabricated (Figure 3c). During stretching, entanglement points slip and induce polymer chains to form a uniformly oriented structure across the entire region. Strain energy is stored via entropy change, achieving nearly 100% mechanical reversibility and low hysteresis [31]. The double-network dynamically crosslinked ionic hydrogel stabilizes conductive pathways through reversible ionic interface reconstruction, greatly attenuating signal drift caused by stretching. It is suitable for high-deformation human wearable scenarios and can simultaneously sense pressure without interference from limb stretching [63]. Furthermore, topological optimization of the conductive network, such as constructing three-dimensional interconnected hierarchical conductive structures, can provide alternative conductive paths when local pathways break, further improving conductive reliability over a wide strain range [44] (see Table 2).

## 5. Typical Application Scenarios

### 5.1. Wearable Epidermal Electronics

Wearable epidermal electronics are conformally attached directly to human skin and must accommodate stretching, bending, and torsional deformation of the skin. Strain-insensitive hydrogels serve as core materials for achieving high-fidelity physiological monitoring [65,66,67].

For physiological electrical signal acquisition, strain-insensitive hydrogel electrodes enable stable recording of electrocardiogram (ECG), electromyogram (EMG), and electrooculogram (EOG) signals under motion conditions. Conventional hydrogel electrodes suffer from drastic impedance fluctuations during limb movement, which readily induce motion artifacts [68]. Shen et al. fabricated a conductive organohydrogel with high lignin content and abundant catechol moieties [69]. After immersion in salt solutions, the gel develops a three-dimensional porous ion transport network, with a resistance increase of only 3% under 100% strain. When used as epidermal electrodes for ECG and EMG recording, it outperforms commercial Ag/AgCl electrodes with respect to the signal-to-noise ratio, enabling high-quality physiological signal acquisition free of motion artifacts. Hydrogel electrodes with stable conductivity can maintain low and steady contact impedance even under large deformation scenarios such as joint bending and muscle contraction, delivering low-noise, high-quality electrophysiological signals suitable for exercise health monitoring [70].

**Figure 4:**
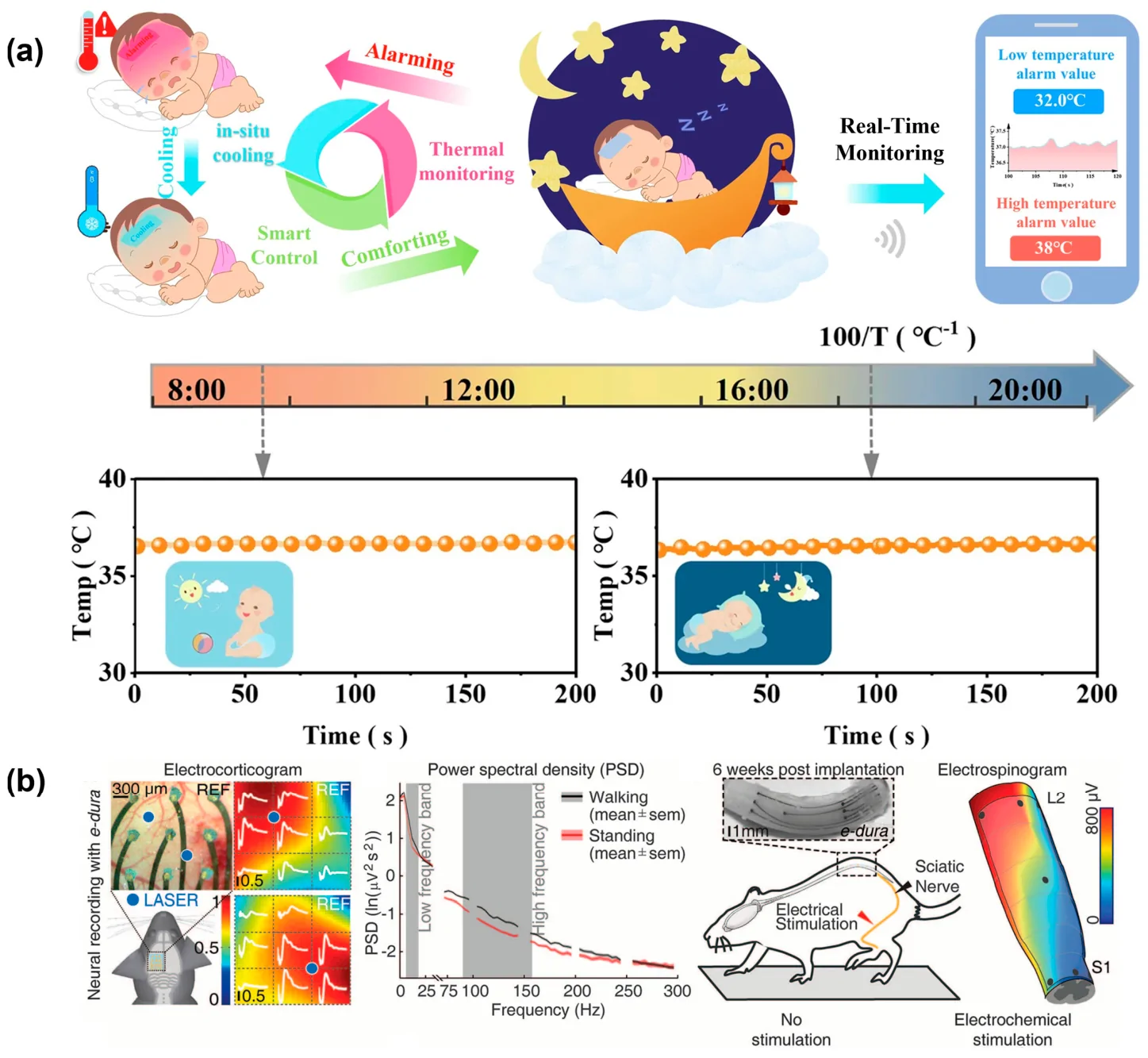
(**a**) A strain-insensitive wearable temperature monitoring patch that integrates an MXene-based flexible thermal sensor with a thermoelectric cooling module for continuous monitoring and in situ cooling therapy. The thermal sensor employs an alternating laminated structure with interfacial interlocking to achieve both high strain insensitivity and high thermal sensitivity. This wearable system enables continuous and precise nocturnal monitoring of infant body temperature. Reproduced with permission: Copyright 2026, Springer Nature [71]. (**b**) The e-dura implant is placed on the cortical surface of a mouse; the blue markers denote the laser irradiation sites. The cortical activation map, shown in white, is reconstructed from normalized electrocorticography (ECoG). Power spectral density was calculated from motor cortical electrophysiological recordings of rats 3 weeks after e-dura implantation. Enhanced neural activities in low- and high-frequency bands can distinguish cortical states during walking and standing. The spinal cord activation map is reconstructed from spinal cord electrogram recordings upon stimulation of the left sciatic nerve in rats 6 weeks after e-dura implantation. Reproduced with permission: Copyright 2015, American Association for the Advancement of Science [77].

In the field of human motion monitoring, strain-insensitive hydrogels act as stable signal transmission lines and reference electrodes (Figure 4a), which can be integrated with strain-sensitive sensing units to construct multimodal sensing systems [71]. Li et al. prepared LiBr-regulated double-dynamic crosslinked conductive hydrogels via a “less but better” synergistic strategy, achieving a gauge factor as low as 0.29 and electrical hysteresis of only 0.19% within 150% strain [72]. Meanwhile, LiBr endows the material with humidity-adaptive water retention and low-temperature tolerance. It maintains conductive stability after 10,000 stretching cycles and half a year of humidity-varying aging, and it can serve as a drift-free conductive interconnection layer for pulse sensing, flexible circuits, and manipulator signal transmission. Signal transmission through strain-insensitive conductive pathways avoids signal interference caused by deformation of the transmission lines themselves, improving the accuracy of motion gesture recognition and physiological parameter detection [73]. Furthermore, favorable skin adhesion and biocompatibility enable long-term wearable use, meeting the demands of daily health management. Luo et al. greatly enhanced the adhesion between hydrogels and metal electrodes and reduced interfacial impedance via electrochemically induced interlocked topological interfaces. The artificial skin fabricated with this system realizes simultaneous multimodal detection of temperature, humidity, and oxygen free of strain interference, effectively suppressing motion artifacts caused by skin stretching [74].

### 5.2. Implantable Bioelectronics

Implantable bioelectronics require long-term residence in the body, accompanied by continuous pulsation and relaxation of the heart, blood vessels, and visceral organs. Devices are exposed to a persistent dynamic strain environment, and strain-insensitive properties directly determine the long-term reliability and biosafety of implantable devices [75].

In the field of neuromodulation and neural interfaces, hydrogel electrodes can form a compliant mechanical interface with neural tissue, while strain-insensitive design ensures stable electrode impedance during organ movement [76]. For example, hydrogel electrodes for neuromodulation exhibit an impedance increase of less than 20% at 1 kHz (10%) and only 3.6% at 1 Hz (20%) under 20% tensile strain, and the impedance remains stable after 10,000 cyclic stretches. This property enables the electrodes to deliver precise electrical stimulation and signal acquisition even at the surface of dynamic organs such as the heart and brain tissue, avoiding degradation of stimulation accuracy and signal distortion caused by impedance drift [15].

In implantable sensing and theranostic devices, strain-insensitive hydrogels, as stable encapsulation materials and conductive media, can shield mechanical interference caused by in vivo tissue motion and preserve the detection accuracy of sensors (Figure 4b) [77]. Zhao et al. proposed a layered composite strain-insensitive bioelectrode (SIB) structure using metal oxide interfacial thin films [78]. Under 150% stretching, the electrochemical impedance and voltammetric curves show almost no shift, enabling stable multi-index sensing of hydrogen peroxide, acetaminophen, and pH, as well as in vivo neuromodulation. However, for long-term implantation, the biosafety of metallic components in the materials remains to be confirmed. Implantable devices should simultaneously take into account conductive stability, adhesion, dehydration resistance, and biocompatibility. Li et al. developed an elastomer–hydrogel biphasic integrated implantable epidermal bioelectronic platform, achieving molecular-level covalent bonding between the elastic conductive substrate and the tissue-adhesive ionic hydrogel via in situ polymerization [79]. The hydrogel buffers mechanical disturbances through dynamic ionic crosslinks, largely eliminating motion artifacts caused by skin stretching and wrinkling. It can stably record multiple types of physiological signals, including ECG, electrochemical glucose, and neural stimulation, enabling real-time physiological signal monitoring and autonomic nerve therapeutic intervention. Meanwhile, the tissue-mimetic mechanical properties of hydrogels can reduce foreign body reaction, improving the biocompatibility and in vivo service life of implantable devices [72,80].

## 6. Conclusions and Perspectives

Strain-insensitive conductive hydrogels break through the limitation that “performance drift inevitably accompanies deformation” in conventional flexible conductors, providing core materials and technical solutions for the construction of highly stable flexible electronics in dynamic environments. To date, multiple implementation pathways have been established from two dimensions: intrinsic material design and device structural engineering. At the material level, the three major systems—LM composites, interfacial regulation of conductive polymers, and hydrogen-bonding network design—each possess distinct advantages, achieving strain-insensitive conductivity from the perspectives of fluid self-healing, interfacial compatibility, filler bridging, and molecular network stability, respectively. At the device level, three strategies—geometric compensation, mechanical decoupling, and interfacial engineering—act synergistically to guarantee device performance over a wide strain range from multiple dimensions, including structural design, mechanical transmission, and interfacial stabilization.

However, a standardized and unified evaluation indicator system has not yet been established in this field, as summarized in Table 3. Different studies typically select varying strain amplitudes and characterization parameters for testing, making it difficult to conduct cross-system benchmarking and quantitative comparisons of the strain-insensitive performance across various material systems. To address this issue, this work establishes a unified quantitative evaluation framework; tensile sensitivity per unit strain, defined as (ΔR/R_0_)/ε, is adopted as the core evaluation indicator, where a lower value signifies superior strain-insensitive performance of the material. Meanwhile, three auxiliary indicators are introduced—maximum tolerable fracture strain, intrinsic electrical conductivity, and gauge factor (GF, which characterizes the sensitivity of resistance change with respect to strain)—to achieve comprehensive characterization of material performance from three dimensions: deformation tolerance, intrinsic conductivity level, and strain response sensitivity.

Despite remarkable research progress, this field still faces numerous challenges. First, the conductive stability under extremely large strain and long-term cycling remains to be improved, and most materials still exhibit performance degradation after exceeding 500% strain or more than 10,000 cycles [12,41]. Second, it is difficult to achieve synergy between multifunctional integration and strain insensitivity; the introduction of functionalities such as self-healing, self-powering, and responsiveness tends to disrupt the stability of the conductive network [81]. Third, scalable fabrication and long-term in vivo biosafety remain to be verified, and there is still a gap in clinical translation and industrial application [78]. Future research should develop intrinsically strain-insensitive materials with multi-mechanism synergy, combining dynamic chemical bonds and topological network design to achieve performance stability over an ultra-wide strain range. Design theories for strain insensitivity under multi-field coupling should be established to guide the precise optimization of materials and devices. Efforts should also be made to advance biosafety evaluation and scalable fabrication process development of material systems, accelerating their practical deployment in wearable healthcare, implantable theranostics, and other related fields.

## Figures and Tables

**Table 1:** Strain-insensitive mechanisms and limitations of typical hydrogel composite materials.

Material Platform	Primary Stabilization Mechanism	Typical Functions	Advantages	Main Limitations
LMs	Flow-enabled self-healing, solid–liquid synergy	Stretchable conductors, flexible electrodes [26]	Self-healable conductive pathways	Leakage, oxidation
Conductive polymers	Molecular entanglement, wrinkle unfolding	Bioelectrodes, bioelectronic interfaces [49]	Low interfacial impedance, stable conductivity	Delamination of conductive, susceptibility to phase
Hydrogen-bonded/isotropic hydrogel	Dynamic bond reconfiguration, isotropic network	Artifact-resistant sensing, skin interfaces [6]	Tunable mechanics, tissue matching, strong adhesion	Temperature–humidity sensitivity

**Table 2:** Design logic, metrics and examples of three categories of solution strategies.

Strategy	Core Objective	Common Designs	Evaluation Metrics	Representative Examples
Geometric and functional compensation	Counteract strain interference	Wrinkled, pre-strain, functional compensation	ΔR/R_0_, cancellation efficiency, signal crosstalk level	Wrinkled dual-modal sensor [51]
Mechanical decoupling and strain isolation	Reduce strain in functional regions	Structural design and modulus zoning	Signal stability, isolation efficiency	Layered composite electrode [59]
Interfacial engineering and network stabilization	Stabilize conductive pathways and interfaces	Dynamic bond interfaces, double-network crosslinking	Cyclic stability, conductivity retention rate	Layered ionic hydrogel [63]

**Table 3:** Quantitative performance comparison of representative strain-insensitive conductive hydrogels.

Reference	Material System	Core Strategy	Conductivity	Max Tolerable Strain	GF	ΔR/R_0_	Cycling Stability
[15]	PEDOT:PSS hydrogel ECH	Hydrogel-elastomer lamination	47.4 S/cm	20%	—	3.6% @10%	10,000 cycles
[23]	Ag nanosheet/amphoteric copolymer hydrogel	Dynamic H-bonding	>1.6 × 10^5^ S/m	500%	—	—	3000 cycles
[26]	LM/hydrogel	Interfacial fusion	1.18 × 10^6^ S/m	>400%	—	~4%	1000 cycles @100%
[48]	PPy/PEDOT	Multiscale interfacial confinement	3.58 S/cm	~200%	~0.18	~18%	—
[52]	HPC/PVA cellulosic material	Isotropic network	—	50%	—	—	3000 cycles
[63]	PVA/CNF eutectogel PCE6	Directional freezing	0.88 S/m	~150%	0.11	7.2%	100 cycles @80%
[55]	PEDOT:PSS/PVA	IPN/helical geometry	147 S/cm	500%	~0.05	5%	2000 cycles
[69]	Lignosulfonate ionogel	Anisotropic adhesion	3.87 × 10^−2^ S/cm	590%	~0.03	3%	5000 cycles @100%
[78]	AgNW/metal-film-layered SIB	Strain isolation	~1 Ω/sq	>150%	—	—	>10,000 cycles

## Data Availability

No new data were created or analyzed in this study. Data sharing is not applicable to this article.
